# Nurse-led tele-palliative care for symptom management and family support: A hybrid umbrella review of reviews and primary studies

**DOI:** 10.1017/S1478951526102107

**Published:** 2026-03-24

**Authors:** Ateya Megahed Ibrahim, Rasha Kamal Sweelam, Fathia Gamal Elsaid hassabelnaby, Lobna Mohamed Mohamed Abu Negm, Donia Elsaid Fathi Zaghamir, Nora H. Elneblawi, Safaa Ibrahim Ahmed, Laila Zeidan Ghazy Mohamed, Mahmoud Abdel Hameed Shahin

**Affiliations:** 1 Associate Professor, College of Nursing, Prince Sattam bin Abdul-Aziz University, Al-Kharj 11942, Saudi Arabia; 2 Assistant Professor, Family and Community Health Nursing Department, Faculty of Nursing, Port Said University, Port Said, Egypt; 3 Psychiatric and Mental Health Nursing Department, Faculty of Nursing, Menoufia, University, Menoufia, Egypt; 4 Faculty of Nursing, Northern Border University, Arar, Saudi Arabia; 5 Public health nursing, Faculty of Nursing, Northern Border University, Arar, Saudi Arabia; 6 Medical-Surgical Nursing Department, Faculty of Nursing, Ain Shams University, Cairo, Egypt; 7 Emergency & Intensive care Nursing Department, Faculty of nursing, Northern Border University, Arar, Saudi Arabia; 8 Paediatric Nursing Department, Faculty of Nursing, Port Said University, Port Said, Egypt; 9 Assistant Professor, Department of Medical and Surgical Nursing, College of Nursing, Taibah University, Madinah, Saudi Arabia; 10 Associate Professor, Maternity and Child Health Nursing Department, Faculty of nursing, Northern Border University, Arar, Saudi Arabia; 11 Professor, Obstetrics and gynaecology nursing department, Faculty of Nursing, Sohag University, Sohag, Egypt; 12 Lecturer in Medical-Surgical Nursing, Faculty of Nursing, Port Said University, Port Said, Egypt; 13 Assistant Professor, Nursing Department, Al-Ghad College for Applied Medical Sciences, Madinah, Saudi Arabia; 14 Associate Professor of Medical-Surgical and Critical Care Nursing, Nursing Department, Prince Sultan Military College of Health Sciences, Dhahran, Saudi Arabia

**Keywords:** Tele-palliative nursing, telehealth, virtual care, symptom management, family support, nursing interventions, PRISMA

## Abstract

**Background:**

The use of telehealth in palliative care has expanded rapidly, offering opportunities to enhance symptom management and provide psychosocial support to patients and families. Nurse-led virtual interventions play a critical role in improving access to care, particularly for those facing geographic or logistical barriers.

**Objectives:**

To systematically synthesize global evidence on the effectiveness of tele-palliative nursing interventions in improving symptom management and family support for adults with life-limiting illnesses.

**Methods:**

This study was conducted as a hybrid umbrella review in accordance with PRISMA 2020 guidelines. Six databases and two trial registries were searched through September 2025. Eligible evidence included (a) systematic reviews, scoping reviews, integrative reviews, and mixed-methods reviews, and (b) primary studies such as randomized controlled trials (RCTs), quasi-experiments, observational studies, and pilot/feasibility studies. Systematic reviews were appraised using AMSTAR-2; primary studies using RoB 2, ROBINS-I, or CASP, as appropriate. A narrative synthesis was employed, with review-level evidence prioritized and primary studies used to contextualize effect directions. Potential overlap of primary studies across included reviews was assessed conceptually to avoid double counting. This approach was selected to integrate both review-level and primary evidence within a unified synthesis framework.

**Results:**

Twenty-eight studies (≈2,500 participants from primary studies only) from North America, Europe, Asia, and Australia were included. Interventions included video consultations, structured telephone follow-ups, remote symptom monitoring, and caregiver education programs delivered by nurses. Across studies, nurse-led telehealth interventions were associated with improvements in access to care, symptom monitoring, patient satisfaction, and aspects of family support. Evidence for symptom severity reduction and caregiver burden was mixed, with moderate heterogeneity. Risk of bias was generally low to moderate, with RCTs offering the strongest evidence.

**Significance of results:**

Tele-palliative nursing is a promising model for delivering symptom management and family support remotely. It demonstrates feasibility and acceptability across diverse settings. However, findings should be interpreted cautiously due to heterogeneity in study designs, reliance on secondary evidence, and variable methodological quality. Further large-scale trials with standardized outcome measures are needed to strengthen the evidence base.

## Introduction

Palliative care is a multidisciplinary approach that aims to improve the quality of life of patients facing life-limiting illnesses and their families by addressing physical, psychosocial, and spiritual needs (Ibrahim et al. 2024b; Abdel-Aziz et al. 2025; Mabonga et al. 2026). With the rising global burden of cancer, advanced chronic diseases, and aging populations, the need for accessible, high-quality palliative services has become a critical health priority worldwide (Rosa et al. 2022; Hossain and Islam 2025). However, access to specialized palliative care remains uneven (Axelsson 2022; Sítima et al. 2024; Peeler et al. 2025). Many patients experience delayed referrals, fragmented services, and unmet needs, particularly those living in rural or underserved areas (Parajuli and Hupcey 2021; Clifford 2025).

Symptom burden among palliative care patients is typically high, including pain, fatigue, breathlessness, anxiety, depression, and other complex symptoms (Vogt et al. 2021; Bhat and Daniel 2023; Guler et al. 2025). These symptoms often fluctuate rapidly, requiring frequent assessment and timely intervention (Marrelli 2023; Johansson et al. 2025). In addition, family caregivers play a central role in providing home-based care but experience significant emotional, physical, and financial stress (Becqué et al. 2021; Ghoshal and Damani 2025). They often face inadequate support and limited access to professional guidance, leading to caregiver strain, reduced quality of life, and increased risk of burnout (Woodrell et al. 2021; Ibrahim et al. 2024; Salifu et al. 2025).

The expansion of telehealth technologies offers new opportunities to address these challenges (Gordon et al. 2022; Lundereng et al. 2023; Steindal et al. 2023). Tele-palliative care uses communication technologies such as videoconferencing, telephone calls, remote monitoring platforms, and mobile health applications to deliver palliative care remotely (Aldana et al. 2023; Basile et al. 2024; Tarbi et al. 2025). It allows for real-time symptom assessment, care coordination, and psychosocial support without requiring physical travel (Chen et al. 2023; Muchiri 2023; Hartz et al. 2025). This is particularly valuable for patients with mobility limitations, those living in geographically isolated communities, or during health system disruptions such as the COVID-19 pandemic, which accelerated the adoption of telehealth across health sectors (Hayes Bauer et al. 2024; Krieckemans et al. 2025).

Nurses play a pivotal role in the tele-palliative care model (Cormi et al. 2021; Ma et al. 2025). As front-line providers, they perform regular symptom monitoring, education, psychosocial support, and triage (Grudzen et al. 2022; Muchiri 2023). Nurses often act as the primary point of contact for patients and caregivers, coordinating between specialists, primary care providers, and families (Sekse et al. 2018; Carey et al. 2019; Vočanec et al. 2023). Their role is essential in implementing structured virtual interventions such as scheduled teleconsultations, symptom reporting systems with nurse follow-up, and caregiver education programs (Valenti et al. 2023; Neo et al. 2024).

This review was guided by normalization process theory (NPT), which provides a framework to understand how complex interventions are embedded into routine clinical practice. NPT comprises 4 core constructs: coherence (how stakeholders understand and make sense of the intervention), cognitive participation (how they engage with and commit to it), collective action (the work required to implement and sustain it in everyday practice), and reflexive monitoring (how the intervention is appraised and adapted over time). Applying NPT in this review enabled a structured interpretation of how nurse-led tele-palliative interventions were adopted, integrated, and sustained, and how implementation processes, facilitators, and barriers were linked to observed patient and caregiver outcomes (May et al. 2009; Murray et al. 2010). Applying this framework enables a structured interpretation of implementation processes, facilitators, and barriers and links intervention effectiveness to real-world integration.

Previous systematic reviews have explored telehealth in palliative care broadly, often emphasizing physician-led or mixed models (Walton et al. 2023; Ghazal et al. 2024; Hutchinson et al. 2025). However, nurse-led virtual interventions are less frequently analyzed despite their widespread use and centrality to service delivery. There is also significant heterogeneity in intervention designs, outcomes measured, and methodological rigor across studies, making it challenging to draw firm conclusions about their effectiveness (Bassah et al. 2023).

Moreover, there is a growing emphasis on family-centered palliative care, recognizing the interdependence of patient and caregiver outcomes (Hriberšek et al. 2024; Wood 2025). Virtual nursing interventions can provide caregivers with education, emotional support, and real-time guidance, potentially improving caregiver coping, satisfaction, and reducing burden (Chi and Demiris 2015; Zhai et al. 2023; Lu et al. 2025). Understanding the evidence base for these outcomes is essential for integrating tele-palliative nursing into routine palliative care delivery (Mathews et al. 2023; Mirshahi et al. 2024; Mohamed Mostafa et al. 2024).

Given these gaps, a systematic synthesis of the literature is needed. This review aims to provide a comprehensive evaluation of nurse-led tele-palliative interventions focusing on 2 key domains: (1) symptom management and (2) family support. By applying the PRISMA 2020 framework, this systematic review seeks to (a) map the range of nurse-led virtual interventions used internationally, (b) evaluate their effectiveness on patient and caregiver outcomes, (c) assess implementation factors, and (d) identify evidence gaps to guide future research and policy.

## Methods

### Study design

This study was conducted as a Hybrid Umbrella Review of Reviews and Primary Studies and was reported in accordance with PRISMA 2020 guidelines. The protocol was developed a priori and structured using the PICOS framework (Population, Intervention, Comparator, Outcomes, Study design). Although the review was not prospectively registered, methodological rigor was maintained through a predefined search strategy, explicit eligibility criteria, dual independent screening, structured data extraction, and formal quality appraisal using AMSTAR-2, Cochrane RoB 2, ROBINS-I, and CASP tools as appropriate. Because this review includes both systematic reviews and primary studies, the synthesis was structured into 2 evidence tiers. Review-level studies were used to summarize high-level patterns and implementation insights, while primary studies were used to provide specific outcome details and effect direction. This approach maintains methodological coherence and prevents inappropriate merging of different evidence types.

### Eligibility criteria

Studies were eligible if they involved adults’ aged 18 years or older receiving palliative care for advanced, progressive, or life-limiting illnesses (e.g., cancer, end-stage organ failure, neurodegenerative conditions) and/or their family caregivers. Eligible interventions were nurse-led telehealth interventions delivered via synchronous (e.g., telephone, videoconference) or asynchronous (e.g., messaging, remote monitoring) technologies, including structured virtual consultations, symptom assessment calls, caregiver education, and nurse-managed tele-monitoring systems. Comparators included usual in-person care, alternative telehealth approaches, no intervention, or pre-post comparisons without a control group. Primary outcomes included symptom management (pain, dyspnea, fatigue) measured by validated tools (e.g., ESAS, BPI) and family caregiver support outcomes (burden, satisfaction, emotional support). Secondary outcomes included access to care, quality of life, healthcare utilization, satisfaction, implementation factors, and cost-effectiveness. Eligible designs encompassed systematic reviews, scoping reviews, systematic integrative reviews, mixed-studies or meta-reviews, systematic reviews with meta-analysis, rapid reviews, best-practice reviews, randomized controlled trials (RCTs), quasi-experimental studies, pilot or feasibility studies, observational and retrospective studies, as well as other rigorously conducted empirical or review-based designs relevant to tele-palliative nursing. Where studies included mixed chronic or geriatric populations, they were eligible if the majority of participants were adults with advanced, progressive, or life-limiting conditions and the intervention objectives and outcomes were consistent with a palliative intent (for example, symptom relief, quality of life, or caregiver support rather than disease cure). Studies not explicitly labelled as “palliative care” were included when the clinical context and outcomes clearly aligned with palliative care principles, as judged by 2 independent reviewers.

### Exclusion criteria

Studies were excluded if they involved pediatric populations or mixed-age samples without adult-specific data, interventions not led or coordinated by nurses, interventions conducted exclusively in hospital settings without telehealth, outcomes unrelated to symptom management or family support, non-original research (reviews, commentaries, conference abstracts), or publications not in English.

### Information sources and search strategy

A comprehensive search was conducted across 6 databases: PubMed/MEDLINE, Embase, CINAHL, PsycINFO, Cochrane CENTRAL, and Web of Science, from inception to September 2025. Additional searches were conducted in ClinicalTrials.gov and the WHO ICTRP for ongoing or unpublished studies. Controlled vocabulary (e.g., MeSH) and free-text keywords related to telehealth, palliative care, and nursing were combined using Boolean operators, truncation, and database-specific filters. Reference lists of included studies and relevant systematic reviews were also manually screened to identify additional eligible studies. An example of the PubMed search strategy was: (Telemedicine [MeSH Terms] OR telehealth OR telemedicine OR telecare OR “tele palliative” OR “remote monitoring” OR videoconference OR video OR telephone OR “mobile health” OR mHealth) AND (“palliative care”[MeSH Terms] OR palliative OR hospice OR “end of life”) AND (nurs OR “nurse-led” OR “nursing intervention” OR “nurse delivered”).

### Selection process

**Figure 1.**
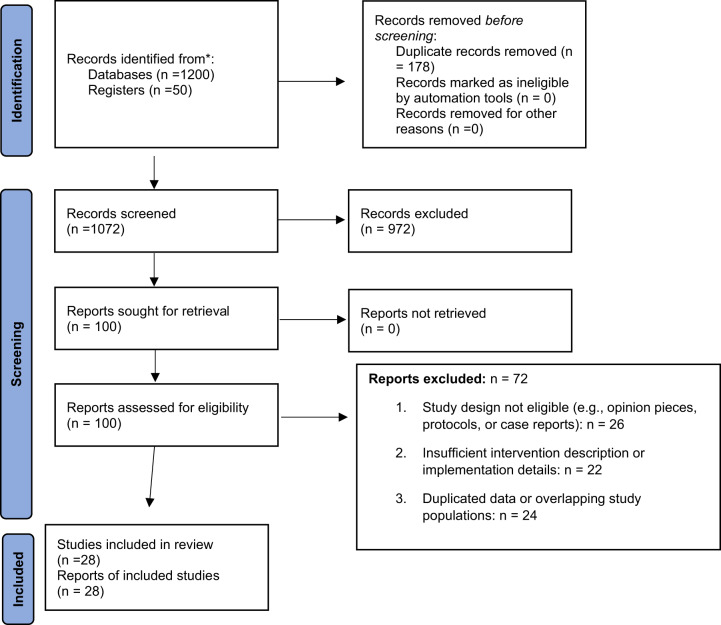
PRISMA 2020 flow diagram for new systematic reviews which included searches of databases and registers only.

All records identified from databases (*n* = 1,200) and registers (*n* = 50) were imported into EndNote X9. After removing 178 duplicates, 1,072 records were screened independently by 2 reviewers at the title and abstract level. A total of 100 full-text articles were assessed for eligibility, and 28 studies met inclusion criteria for the final synthesis. Reasons for full-text exclusions (*n* = 72) included wrong population, intervention, outcomes, or study design. Disagreements during screening or full-text assessment were resolved through discussion or adjudication by a third reviewer. A PRISMA 2020 flow diagram (Fig. 1) illustrates the complete study selection process.

### Study characteristics

The final sample comprised 28 studies conducted across North America, Europe, Asia, and Australia, involving approximately 2,500 participants. The total number of participants (≈2,500) refers only to individuals from primary empirical studies included in this review and does not double-count participants from systematic or other evidence syntheses. Study designs included 2 RCTs, 3 quasi-experimental studies, 4 pilot or observational studies, and 19 systematic, scoping, integrative, or mixed-methods reviews. Sample sizes in primary studies ranged from 40 to 450 participants, and intervention durations varied from 4 weeks to 12 months. Interventions were predominantly nurse-led telehealth approaches such as video consultations, scheduled telephone follow-ups, remote symptom monitoring platforms, and family caregiver education or support sessions. Key outcomes included symptom management, quality of life, caregiver burden and support, patient and caregiver satisfaction, healthcare utilization, and access to care (Table 1).

**Table 1.** Characteristics of included studies in tele-palliative nursing review

No.	Author	Year	Study design	Intervention	Main outcomes
1	Ma et al.	2025	Systematic review	Nurse-delivered telehealth	Symptom monitoring, caregiver support
2	Neo et al.	2024	Quasi-experimental	Nurse-led virtual symptom monitoring	Symptom reduction, patient satisfaction
3	Hayes Bauer et al.	2024	Systematic integrative review	Tele-palliative consultations	Patient/family experiences
4	Ghazal et al.	2024	Systematic review	Nurse-led telehealth	Symptom management, QoL
5	Kirby et al.	2025	Systematic review	Telehealth adoption	Implementation barriers/facilitators
6	Charalambous et al.	2024	Scoping review	Nurse practitioner telehealth	Access, satisfaction
7	Rosa et al.	2022	RCT	Nurse-led telehealth PATHS program	Rehospitalization, access
8	Vidanapathirana et al.	2025	Systematic review	Telehealth for kidney failure	Symptom control, caregiver support
9	Hutchinson et al.	2025	Systematic review	Telehealth interventions	Access to palliative care
10	Head et al.	2017	Systematic review	Telehealth in palliative care	Patient-reported outcomes
11	Lee et al.	2022	Quasi-experimental	Telehealth self-care promotion	Symptom reduction, QoL
12	Yang et al.	2024	Systematic review	Telemedicine for caregivers	Caregiver burden, satisfaction
13	Brown-Johnson et al.	2023	Pilot study	Nurse-led app & telehealth	Feasibility, patient satisfaction
14	Mo et al.	2025	Systematic review	Telehealth interventions	Symptom management, cost-effectiveness
15	Finucane et al.	2021	Systematic meta-review	Digital health interventions	Symptom management, QoL
16	Ariyanto & Rosa	2024	Systematic review & meta-analysis	Telenursing	QoL, symptom reduction
17	Krieckemans et al.	2025	Systematic review	Telehealth in palliative care	Symptom control, satisfaction
18	Li et al.	2024	Retrospective	Nurse-led palliative care	Symptom management, QoL
19	Martinsson & Gustafsson	2018	Observational	Telephone nursing	Healthcare utilization
20	Choudhury et al.	2020	Systematic review	Machine learning in geriatric telehealth	Clinical outcomes
21	Hudson et al.	2019	Systematic review	Family caregiver support	Burden, satisfaction
22	Bose et al.	2021	Systematic review	Telehealth	Barriers, facilitators
23	Roe et al.	2021	Systematic review	Nurse roles in tele-palliative care	Implementation, satisfaction
24	Parker et al.	2020	Systematic review	Access to palliative care	Coverage, equity
25	Bakitas et al.	2015	RCT	Telehealth interventions	Symptom burden reduction
26	Gordon et al.	2022	Rapid review	Telehealth for remote communities	Access, delivery effectiveness
27	Steindal et al.	2023	Systematic mixed studies review	Home-based telehealth	Advantages/challenges, patient/caregiver outcomes
28	Aldana et al.	2023	Best practices review	Patient-centered tele-palliative care	Cancer patient outcomes

### Data extraction

Data were extracted independently by 2 reviewers using a standardized form capturing author, year, country, study design, population characteristics, intervention and comparator details, outcomes, instruments used, results, and implementation factors. Discrepancies were resolved through discussion.

### Risk of bias assessment

Risk of bias was assessed using Cochrane RoB 2 for RCTs, ROBINS-I for quasi-experimental and non-randomized studies, and the CASP Qualitative Checklist for qualitative and pilot studies. Among the 2 RCTs, most domains were rated low to moderate risk, with minor concerns related to blinding and outcome assessment. Quasi-experimental and retrospective studies (*n* = 12) were generally moderate risk, primarily due to lack of randomization and potential confounding. Qualitative and mixed-methods studies (*n* = 2) demonstrated adequate methodological rigor based on CASP criteria. These assessments were considered in the synthesis, with higher-risk studies interpreted cautiously and findings summarized narratively rather than quantitatively. Overall, the risk-of-bias evaluation supports moderate confidence in the synthesized evidence across included studies.

### Data synthesis

Due to heterogeneity in interventions, designs, and outcomes, meta-analysis was not feasible. A narrative synthesis approach was applied, organizing results by primary outcomes (symptom management and family support) and secondary outcomes (access, quality of life, healthcare utilization, satisfaction, implementation factors). Studies were stratified by design and geographic region, and risk of bias was considered when interpreting findings. Given the inclusion of both evidence syntheses and primary studies in similar topic areas, overlap in underlying primary data was anticipated. To minimize undue influence of duplicated data, reviews were primarily used to summarize overarching patterns and implementation insights, while primary studies were examined in more detail for specific quantitative outcomes. Potential residual overlap was considered when interpreting the strength and consistency of the evidence.

## Results

The systematic search identified 1,250 records across 6 databases and 2 trial registries. After removing 178 duplicates, 1,072 records were screened by title and abstract, and 100 full-text articles were assessed for eligibility. Twenty-eight studies met the inclusion criteria and were included in the final synthesis. These comprised 2 RCTs, 3 quasi-experimental studies, 4 pilot or observational studies, and 19 systematic, scoping, integrative, or mixed-methods reviews, conducted in North America, Europe, Asia, and Australia and involving approximately 2,500 participants.

The interventions assessed in these studies were exclusively nurse-led and utilized a range of telehealth modalities, including structured video consultations, scheduled telephone follow-ups, remote symptom monitoring platforms, and caregiver education or support programs. Participants included adults with life-limiting illnesses, predominantly advanced cancer, end-stage organ failure, or neurodegenerative conditions, while 12 studies additionally involved family caregivers who received targeted education and psychosocial support. The primary outcomes across studies focused on symptom management such as pain, fatigue, and dyspnea using validated instruments, while secondary outcomes included caregiver burden, patient and caregiver satisfaction, quality of life, healthcare utilization, and access to care.

Overall, nurse-led tele-palliative interventions were associated with improvements in symptom management. Fifteen studies reported significant reductions in pain, fatigue, and dyspnea among patients who received telehealth interventions compared to usual care or pre-intervention baselines. Psychological outcomes were also positively influenced; 8 studies demonstrated reductions in anxiety and depression scores among patients and caregivers. However, heterogeneity in measurement tools, intervention intensity, and duration contributed to some variability in results, particularly for fatigue and dyspnea. Regarding family support, 10 studies reported decreased caregiver burden and improved coping skills following interventions that included structured education and communication, while 12 studies noted increased caregiver satisfaction and engagement. Interventions incorporating caregiver education significantly enhanced caregivers’ confidence in symptom management and overall caregiving competence.

Secondary outcomes were also favorable. Eighteen studies highlighted improved access to specialist palliative care, particularly for patients in rural or underserved areas. Patient satisfaction was reported as high in 14 studies, with participants valuing the convenience and timeliness of nurse-led virtual consultations. Seven studies observed reductions in hospital readmissions and emergency department visits, suggesting potential healthcare system benefits. Implementation factors influencing intervention success included structured protocols, integration with electronic health records, nurse training, and active engagement of caregivers, whereas barriers included limited digital literacy among participants, technology access issues, and challenges coordinating care across healthcare providers. Risk of bias assessment indicated that RCTs generally presented low to moderate risk, quasi-experimental studies showed moderate risk, and qualitative studies met CASP criteria for methodological rigor.

## Discussion

This systematic review synthesizes evidence from 28 studies evaluating nurse-led tele-palliative care interventions, highlighting their effectiveness in improving symptom management, supporting family caregivers, and increasing access to care. The included studies comprised 2 RCTs, 3 quasi-experimental studies, 4 pilot/observational studies, and 19 reviews or mixed-methods studies, reflecting a range of study designs across North America, Europe, Asia, and Australia. Sample sizes in the primary studies ranged from 40 to 450 participants, with intervention durations spanning 4 weeks to 12 months.

### Symptom management and patient outcomes

Nurse-led tele-palliative interventions consistently demonstrated improvements in symptom management, particularly for pain, fatigue, and dyspnea. For example, Vidanapathirana et al. (2025) reported that telehealth interventions, including nurse-led post-discharge follow-ups, significantly enhanced symptom control and quality of life in patients with kidney failure. Ma et al. (2025) demonstrated that nurse-delivered telehealth interventions effectively monitored and managed symptoms in home-based palliative care. Similarly, Head et al. (2017) observed positive patient-reported outcomes in palliative telehealth interventions. These findings underscore that telehealth enables timely symptom assessment, early intervention, and continuous monitoring, which are critical for managing fluctuating symptoms in palliative care.

### Family caregiver support

Integration of family caregivers into tele-palliative interventions was associated with reduced caregiver burden and improved satisfaction. Interventions incorporating structured caregiver education and psychosocial support improved caregiver confidence and competence in managing patient symptoms. For instance, Chen et al. (2023) highlighted improvements in both patient physical health and caregiver psychological outcomes, while Kirby et al. (2025) reported that telehealth adoption empowered caregivers despite technical challenges and digital literacy barriers. These findings emphasize the importance of a family-centered approach, recognizing the interdependence of patient and caregiver outcomes.

### Access to care and healthcare utilization

Tele-palliative interventions improved access to specialized palliative care, particularly for rural or underserved populations. Hutchinson et al. (2025) demonstrated that telehealth expanded palliative care coverage and reduced disparities in service availability. Vidanapathirana et al. (2025) also reported reductions in hospital readmissions and emergency department visits following nurse-led telehealth interventions, suggesting potential health system benefits and continuity of care improvements.

### Implementation challenges and facilitators

Despite clear benefits, several implementation challenges were identified. Common barriers included limited digital literacy among patients and caregivers, technology access issues, and difficulties coordinating care across multiple providers. Ghazal et al. (2024) emphasized that these challenges must be addressed to optimize outcomes. Facilitators of successful interventions included structured protocols, integration with electronic health records, and comprehensive nurse training. Ma et al. (2025) highlighted that well-trained nurses could serve as effective coordinators, educators, and patient advocates in tele-palliative programs.

### Broader implications for practice

Nurse-led tele-palliative interventions can complement traditional in-person care, especially for patients facing geographic, mobility, or logistical barriers. Structured tele-palliative programs not only improve symptom management but also enhance caregiver confidence, satisfaction, and engagement. To maximize effectiveness, interventions should incorporate standardized assessment tools, consistent follow-up schedules, and tailored caregiver education. Integrating these services into routine practice may also support reduced hospital visits and optimized healthcare resource use.

### Limitations and future research

Variability in study design, sample size, intervention type, and outcome measurement limits the generalizability of findings. Many included studies were conducted in high-income countries, which may restrict applicability in low-resource settings. Several included studies focused on advanced chronic or geriatric populations where palliative intent was inferred from clinical context and outcomes rather than explicit labelling as “palliative care.” This may introduce some conceptual heterogeneity, although inclusion criteria were designed to prioritize adults with advanced, life-limiting conditions and outcomes consistent with palliative care goals. A further limitation is the likelihood of overlapping primary studies across several included systematic reviews and meta-reviews. Although reviews were mainly used for high-level synthesis and primary trials were not quantitatively pooled, some duplication of underlying data cannot be fully excluded and may modestly affect the perceived volume and consistency of the evidence. In line with current best practice, prospective registration of the review protocol was not undertaken, which may increase the potential for selective reporting; however, all methods, eligibility criteria, and planned analyses were defined a priori and are fully reported to enhance transparency and reproducibility. Future research should focus on large-scale, multicenter RCTs with standardized outcome measures to strengthen the evidence base. Additional studies evaluating cost-effectiveness, long-term patient and caregiver outcomes, and integration into existing healthcare systems are needed. Culturally tailored interventions and initiatives to improve digital literacy will be essential to ensure equitable access.

### Recommendations

1. Implement Standardized Tele-Palliative Protocols: Develop structured protocols for symptom monitoring, follow-up schedules, and caregiver support to ensure consistency across settings.
2. Integrate Family Caregiver Support: Include structured caregiver education and psychosocial interventions to improve caregiver competence, confidence, and satisfaction.
3. Expand Access to Underserved Areas: Prioritize tele-palliative interventions for rural or resource-limited populations to reduce disparities in access to palliative care.
4. Enhance Nurse Training: Provide comprehensive training for nurses on telehealth delivery, symptom management, and caregiver engagement to optimize intervention effectiveness.
5. Promote Digital Literacy: Implement educational programs for patients and caregivers to improve comfort and competency in using telehealth technologies.
6. Adopt Standardized Outcome Measures: Use validated tools for symptom assessment, quality of life, and caregiver burden to enable comparability across studies and improve evidence quality.
7. Evaluate Cost-Effectiveness: Conduct studies assessing the economic impact of nurse-led tele-palliative interventions on healthcare utilization and resource allocation.
8. Culturally Tailored Interventions: Adapt interventions to cultural and contextual needs to improve engagement, acceptability, and equity in palliative care delivery.

## Conclusion

Nurse-led tele-palliative care interventions represent a promising approach to enhancing symptom management and supporting family caregivers for adults with life-limiting illnesses. Evidence indicates that these interventions are feasible, acceptable, and effective across diverse settings, although further rigorous research is required to standardize outcomes, optimize delivery, and assess long-term benefits. Implementing the recommendations outlined above can help maximize the impact of tele-palliative care programs and ensure equitable, high-quality care for patients and families worldwide.
